# Two Decades of Fogarty Training: Nurturing Global Health Scientists

**DOI:** 10.5334/aogh.5223

**Published:** 2026-08-19

**Authors:** Celia Wolfman, UnJa Hayes, Roger I. Glass, Peter H. Kilmarx, Rachel Sturke

**Affiliations:** 1 Fogarty International Center, National Institutes of Health, Bethesda, Maryland, USA; 2 CMB Foundation, Cambridge, Massachusetts, USA

**Keywords:** global health, education, medical graduate, career development, international cooperation, research personnel

## Abstract

*Background:* Scientific advances in global health rely on interdisciplinary researchers collaborating across cultures and orders to tackle complex health challenges. Sustained investment in the next generation of researchers is essential to maintaining this critical global health science capacity. This original article examines factors associated with the Fogarty International Center’s (FIC) research training programs that produce independent global health researchers.

*Objectives:* To examine factors within the FIC’s research training programs that are associated with the successful production of independent global health scientists and to evaluate the long-term impact of the programs on alumni careers.

*Methods:* This analysis synthesizes evidence from two cross-sectional surveys of FIC program trainees spanning the years 2003–2021. The surveys collected data from 717 and 690 alumni (with a 58% and 56% response rate, respectively). The analysis incorporates training experiences, career trajectories, and factors that influence professional independence, productivity, and research commitment.

*Findings:* The analysis shows the program has successfully supported and expanded the global health research workforce. Sixty-two percent (62%) of alumni have careers in academia and 59% are actively working in research. Respondents consistently noted the importance of mentoring, protected research time, and robust professional networks contributing to a successful fellowship experience. The program has effectively stimulated interest, enlisted new partners, and addressed workforce development needs in global health research among early-career professionals in public health and medicine.

*Conclusions:* FIC’s research training programs are a successful model for developing the global health research workforce. Sustained training that emphasizes strong mentorship, dedicated research time, and collaborative partnerships is critical for fostering professional development and maximizing the long-term impact on global health equity.

## Background

Training a workforce capable of conducting research in global health is essential to advance our understanding of the many diseases that have an infectious, environmental, or genetic basis and a distinct pattern of distribution across countries and populations. Recent global health events underscore that pandemics transcend national borders and demonstrate the importance of global surveillance and research partnerships in enabling the early detection of emerging threats and expediting the development of effective responses. However, unlike many countries, the United States includes individuals and communities with origins in every region of the world, including populations affected by diseases that may be uncommon or have different epidemiologic patterns in the United States. Strategies to understanding the natural history, diagnosis, treatment, and prevention of these conditions can be advanced most effectively through established collaborations with researchers and communities in countries where these diseases are most prevalent. Similarly, diseases associated with environmental exposures to contaminants in air, water, food, and products may be more readily characterized by taking science to populations experiencing higher or more varied levels of exposure to these conditions. Cross-country variation in the distribution of NCDs, like cancer, provides additional opportunities to investigate complex interactions among environmental exposures, genetic susceptibility, and cultural practices. In short, advancing biomedical research to improved public health requires a workforce prepared to collaborate with global partners, particularly in settings where the diseases, exposures, and conditions under study are most prevalent.

History provides compelling examples of how research conducted in settings with distinct patterns of disease can generate fundamental insights with implications extending far beyond the populations in which those observations were first made. The unusually high incidence and distinctive geographic distribution of a childhood cancer in equatorial Africa [1, 2] led Denis Burkitt, a researcher working in Uganda, to propose an infectious etiology. Subsequent research identified the Epstein–Barr virus (EBV) and established its association with the cancer that now bears his name, Burkitt lymphoma [3]. EBV has since been implicated in several other cancers [4, 5], including nasopharyngeal carcinoma, Hodgkin lymphoma, and some gastric cancers [6–8].

High-quality research that lead to medical discoveries depend on well-trained scientists who can collaborate effectively with colleagues across disciplines, cultures, and national borders [9–11]. Building this expertise requires individuals with both the scientific expertise and interpersonal and cross-cultural skills necessary to develop productive international partnerships. Preparing the next generation of global health researchers, therefore, requires sustained investments in training, research opportunities, and mentorship [12, 13]. Investment in early career investigators both in the United States and around the world is critical to maintaining scientific capacity needed to address current and future global health challenges [14, 15].

To meet this demand, the Fogarty International Center (FIC) inaugurated the Fogarty Fellows and Scholars (F&S) Program that provided a one-year mentored research experience at research centers in low- and middle-income countries (LMICs). The first iteration of this program, FIC-Ellison Overseas Fellowship in Global Health and Clinical Research Training Program (Ellison), targeted rising US third-year medical students who were each paired with a medical resident from the assigned LMIC institution. The trainees collaborated on a research project with mentors at both the LMIC and US institutions. Subsequent renewals of the program every five years brought administrative changes that resulted in noticeable expansions of the program. The Fogarty International Clinical Research Scholars and Fellows Program (FICRS-F)—now led by Vanderbilt University—included an extension in trainee eligibility, resulting in more medical residents and postdoctoral scientists in the health sciences. Subsequent restructuring in 2012 and 2017 as the Fogarty Global Health Program for Fellows and Scholars (Consortia I and II) provided grants to five and six consortia, respectively, each consisting of four to five US university partners.

**Figure 1:**
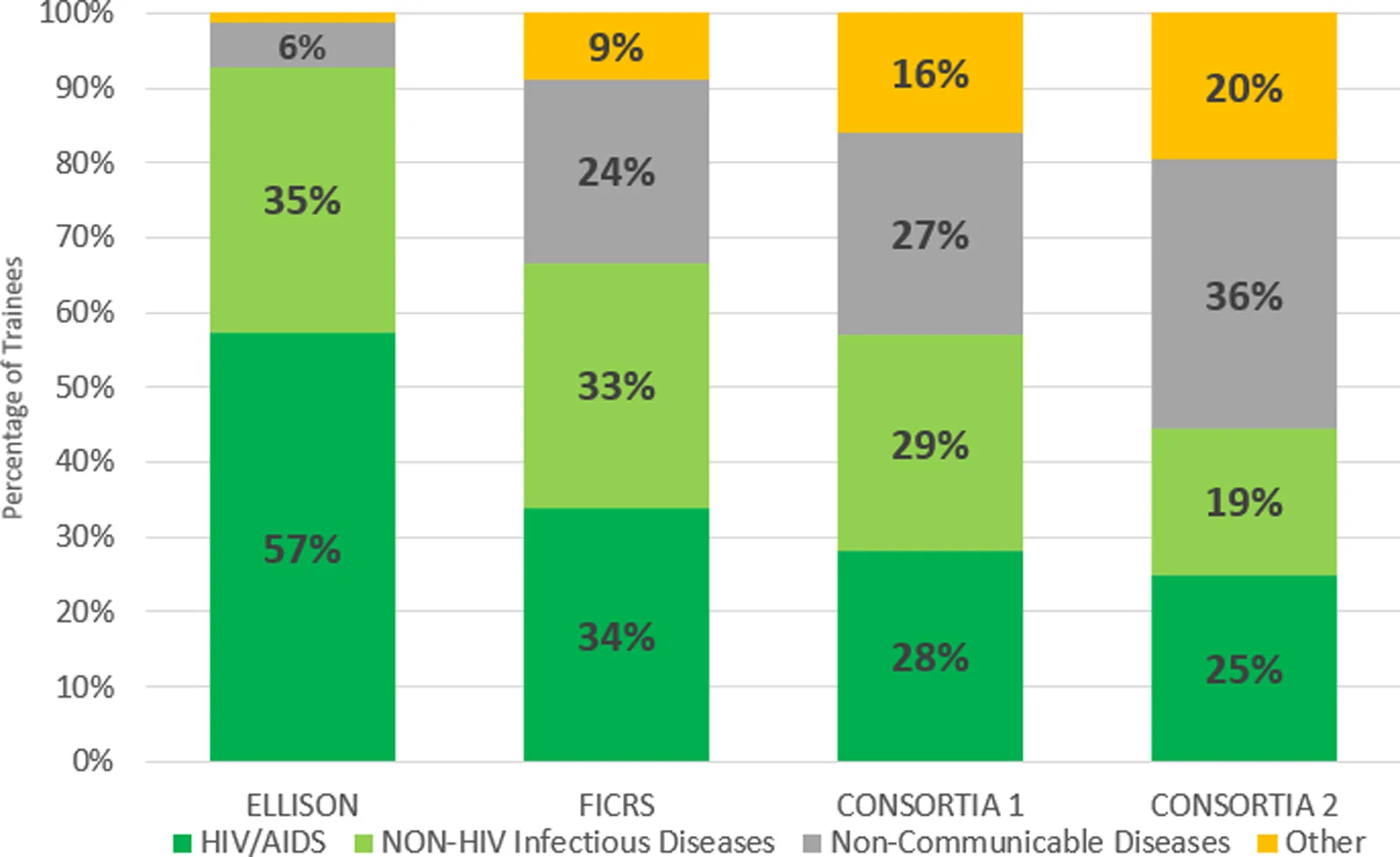
Topical areas of trainee research projects. *Notes*: Others include cross-cutting issues such as maternal or child health, trauma, nutrition, and environmental issues. Co-morbidity research projects may be counted in more than one topical area.

Since inception, the F&S Program nearly doubled in size, going from (on average) 46 predoctoral trainees to 99 pre- and postdoctoral trainees. With that came an expansion of the partner LMICs (15 countries to 39 countries) and the number of funding partners (9 to 19 NIH Institutes, Centers, and Offices ICOs; see Table 1]). The early cohorts of the F&S Program were heavily focused on infectious disease research, including HIV/AIDS (92%; 202; see Figure 1). Fifteen years later, in Consortia II, less than half (44%; 240) of trainee research was focused on infectious disease.

**Table 1:** Evolution of the Fellows & Scholars Program by cycle.

	ELLISON	FICRS	CONSORTIA I	CONSORTIA II
Active Years	2004–2007	2007–2012	2012–2017	2017–2022
Administered Through	Fogarty	Vanderbilt University	Five consortia: GHES, GloCal, NPGH, UJMT, and VECD	Six consortia: GHES, GloCal, NPGH, UJMT, VECD, and HBNU
Total # of Awarded US Universities		1	23	28
# of NIH Institute and Center Funding Partners	9	10	11	19
Yearly average # of Trainees (Total)	46 (185)	75 (376)	85 (427)	99 (493)
# of LMICs	15	24	34	39

An awarded consortium consisted of four partnering US institutions, with some including a US recruitment partner. The first five consortia listed below were awarded in Consortia 1. These awards continued in Consortia II with the addition of the sixth consortia:
**Global Health Equity Scholars Program (GHES):** University of California-Berkeley, Yale University, Stanford University, and University of Arizona). **University of California Global Health Institute Program for Fellows and Scholars (GloCal):** University of California-San Francisco, University of California-Los Angeles, University of California-San Diego, and University of California-Davis, with Charles Drew University. **Northern Pacific Global Health (NPGH):** University of Washington, University of Michigan, University of Minnesota, and Indiana University, with University of Hawaii. **The UJMT Global Consortium: Building Research Capacity through Mentored Training (UJMT):** University of North Carolina at Chapel Hill, Johns Hopkins University, Morehouse School of Medicine, and Tulane University. **Vanderbilt-Emory-Cornell-Duke Consortium for Global Health Fellows (VECDor):** Vanderbilt University, Emory University, Cornell University, and Duke University, with Meharry University. **Partnership for Global Health Research Training Program (HBNU):** Harvard University, Boston University, Northwestern University, University of New Mexico, with University of Puerto Rico.

**Figure 2:**
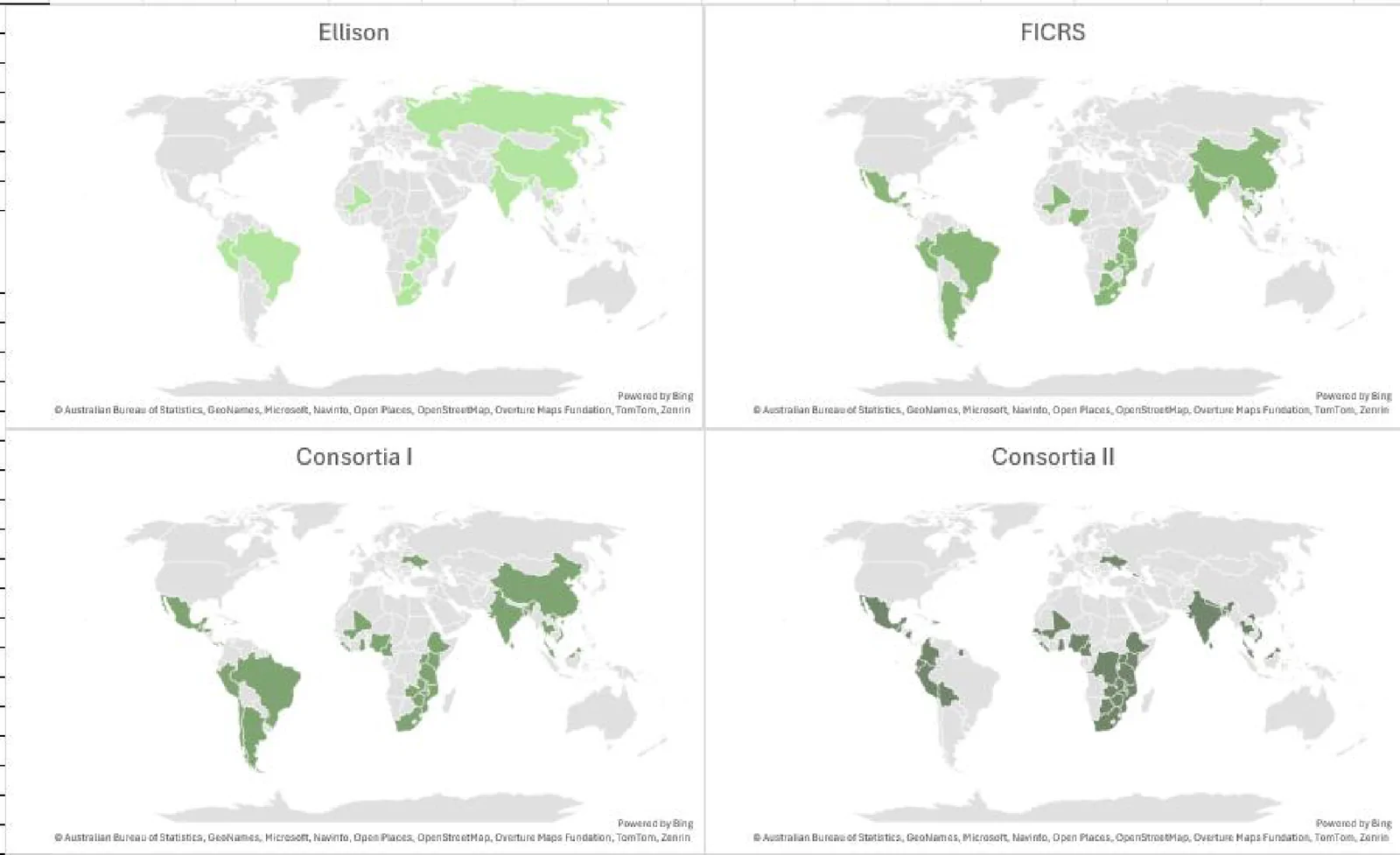
Geographic distribution of program country partnerships across cycles.

The program’s country partnerships across the four program cycles spanned all LMIC regions: Latin America and the Caribbean, Africa, Asia and the Pacific, and Europe, with the most consistent engagement across Africa and Asia and the Pacific (Figure 2). Repeated engagement of countries across cycles was evident in Haiti and Peru in Latin America and the Caribbean; Kenya, Mali, South Africa, Tanzania, Uganda, and Zambia in Africa; and Bangladesh, India, and Thailand in Asia–Pacific. The Elison cycle established foundational countries whose participation was sustained across the cycles, except for those impacted by the policy change restricting participation of G20 countries in Fogarty training programs, namely Brazil, Mexico, Russia, and China (an exception was made for South Africa; NOT-TW-12-01). However, the broadest geographic coverage was seen during Consortia II, with the inclusion of 10 new countries that had not participated in previous cycles: Bolivia, Colombia, Dominican Republic, Ecuador, Nicaragua, and Suriname in Latin America and the Caribbean; the Democratic Republic of the Congo and Senegal in Africa; Nepal and Samoa in Asia and the Pacific; and Georgia in Europe. During Consortia II, a temporary (one-year) exception was granted for Mexico to offset the disruptive impact of the COVID pandemic on training opportunities.

## Objectives

We sought to examine factors within the FIC’s research training programs that are associated with the successful production of independent global health scientists and to evaluate the long-term impact of the programs on alumni careers.

**Table 2:** LMICs participating by cycle.

	ELLISON	FICRS	CONSORTIA I	CONSORTIA II
**LATIN AMERICA & THE CARIBBEAN**				
Argentina		X	X	
Bolivia				X
Brazil	X	X	X	
Chile			X	
Colombia				X
Costa Rica		X		
Dominican Republic				X
Ecuador				X
Guatemala			X	X
Haiti	X	X	X	X
Honduras		X	X	
Jamaica		X	X	
Mexico		X	X	X
Nicaragua				X
Panama			X	
Peru	X	X	X	X
Suriname				X
**AFRICA**				
Botswana	X	X		X
Cameroon			X	X
Democratic Republic of Congo				X
Ethiopia			X	X
Ghana			X	X
Kenya	X	X	X	X
Liberia			X	X
Malawi		X	X	X
Mali	X	X	X	X
Mozambique		X	X	X
Nigeria		X	X	X
Rwanda		X	X	X
Senegal				X
Sierra Leone			X	X
South Africa	X	X	X	X
Tanzania	X	X	X	X
Uganda	X	X	X	X
Zambia	X	X	X	X
Zimbabwe			X	X
**ASIA & THE PACIFIC**				
Bangladesh	X	X	X	X
China	X	X	X	
India	X	X	X	X
Malaysia			X	X
Nepal				X
Samoa				X
Sri Lanka			X	X
Thailand	X	X	X	X
Vietnam		X	X	X
**EUROPE**				
Georgia				X
Russia	X			
Ukraine			X	X

## Methods

Between 2021 and 2022, FIC conducted a program evaluation of the F&S program. As a first step, key FIC staff were consulted to develop a plan for data collection, survey development, and/or analysis. Former grantees who have done similar trainee surveys were consulted in the question development of the survey. Data on award characteristics, mechanism, funding, grantees, funding announcements, and publications were extracted from relevant NIH databases. Information about the first cycle of the program, the Fogarty–Ellison Program, which was not an extramural grant program, was obtained from internal documents as well as Fogarty publications. Available applications and progress reports were manually reviewed for supplemental information. Whenever possible, data were crosschecked across multiple sources to ascertain validity.

To provide robust qualitative evidence on the outcomes and impacts of the F&S Program, trainees were contacted on May 17, 2022, to participate in two separate web-based surveys. The first survey focused on the F&S Program training experience, perceived strengths, and areas for improvement; the second survey focused on post-training accomplishments and employment. Names and emails were obtained from the FIC trainee database, internal tracking spreadsheets, and/or web searches. Given the time span of the program, email addresses could not be obtained for all alumni; 65 individuals could not be contacted in the initial outreach. A good faith attempt was made to find updated email addresses (via inquiries to grantees and/or web searches) for any bounced emails. In the end, emails for roughly 150 individuals (out of 1,477) were unattainable. On July 18, 2022, the surveys were closed after four follow-up requests.

Quantitative data from NIH databases were analyzed in Microsoft Excel. Survey data were captured in SurveyMonkey and exported into Microsoft Excel. Data captured in the surveys were binned into themes manually by FIC analysts using a thematic analysis technique [16]. The general objective of the qualitative assessment was to extract themes to demonstrate the components necessary to build a global health workforce. The themes and narratives were incorporated into a larger document, along with the quantitative data, to provide a larger understanding of the program and identify components that worked or didn’t work in building long-lasting global health careers.

## Findings

The two surveys were distributed via email to 1,477 alumni in July and August of 2022. A total of 717 individuals completed the first survey (58% response rate); these respondents were evenly split between the United States (359; 50%) and LMICs (358; 50%), with slightly more female than male respondents (56% vs. 43%, respectively). Demographics for the respondents of the second survey were very similar. A total of 690 individuals completed the second survey (56% response rate). Again, respondents were nearly evenly split between individuals from the United States (n = 355; 51%) and LMICs (n = 335; 49%), with slightly more female respondents (n = 384; 56%).

When asked about the single most important reason for fellowship success, 60% of respondents cited mentorship, while the remaining responses highlighted institutional infrastructure (11.7%), collaborative networks (9.3%), personal perseverance (5.9%), dedicated funding (5.9%), peers/camaraderie (4.1%), and protected research time (3.4%) as additional contributing factors. This was corroborated by a direct question in which 88.5% of respondents agreed that their mentor played a significant role in their career trajectory, attributing that influence to intellectual guidance and research skill development (53.8% and 52.8%, respectively), career networking and opportunities (44%), and sustained encouragement (28%). Thus, mentorship emerged as the dominant—but not sole—driver of success, operating alongside structural program features and trainees’ own resilience to produce independent global health scientists.

In the same survey, respondents were asked to reflect on challenges they faced during their fellowship. The training cohorts from 2019 to 2021 had to quickly adapt to the evolving disruptions brought on by the COVID pandemic, such as restricted travel, delayed start dates, and scarce in-person interactions. While many people acknowledged that protected time to conduct research was an added benefit of the fellowship, there was a handful of respondents (mostly LMIC trainees) who felt it was challenging to continue meaningful research with so many clinical and administrative demands on their time remaining. Both US and LMIC respondents acknowledged negative experiences pertaining to funding and reimbursements, delays in IRB approval, or lack of in-country administrative support to navigate issues related to travel, housing, legal documents, and visas.

Given that the F&S Program has undergone multiple iterations, several of the challenges identified were addressed through subsequent programmatic revisions. For example, the transition in 2012 to a decentralized, consortium-based model strengthened mentorship and broadened the range of research topics to better reflect the diversity of global health challenges. This approach leveraged established research collaborations between US and LMIC investigators and institutions, creating a stronger foundation for trainee support and research engagement. The restructuring also facilitated a more localized understanding of administrative and operational challenges, enabling the development of strategies to address issues related to funding management and in-country administrative support.

Additionally, from 2004 to 2011, US and LMIC trainees were matched, or “twinned,” based on similar research interests and professional experience. While this model was intended to foster collaboration and peer support, it required substantial administrative investment. Survey findings regarding the twinning experience were mixed. Although a majority of respondents (59%) reported a positive experience, qualitative feedback revealed considerable variability in outcomes, ranging from little or no interaction between matched trainees to the development of enduring professional and personal relationships. These findings suggest that the effectiveness of the twinning strategy depended heavily on how it was implemented and that greater standardization couldhave improved participant experiences. In recognition of these challenges that exceeded available resources, the twinning model was discontinued in the 2012 revision of the F&S Program (Consortia I and II).

**Figure 3:**
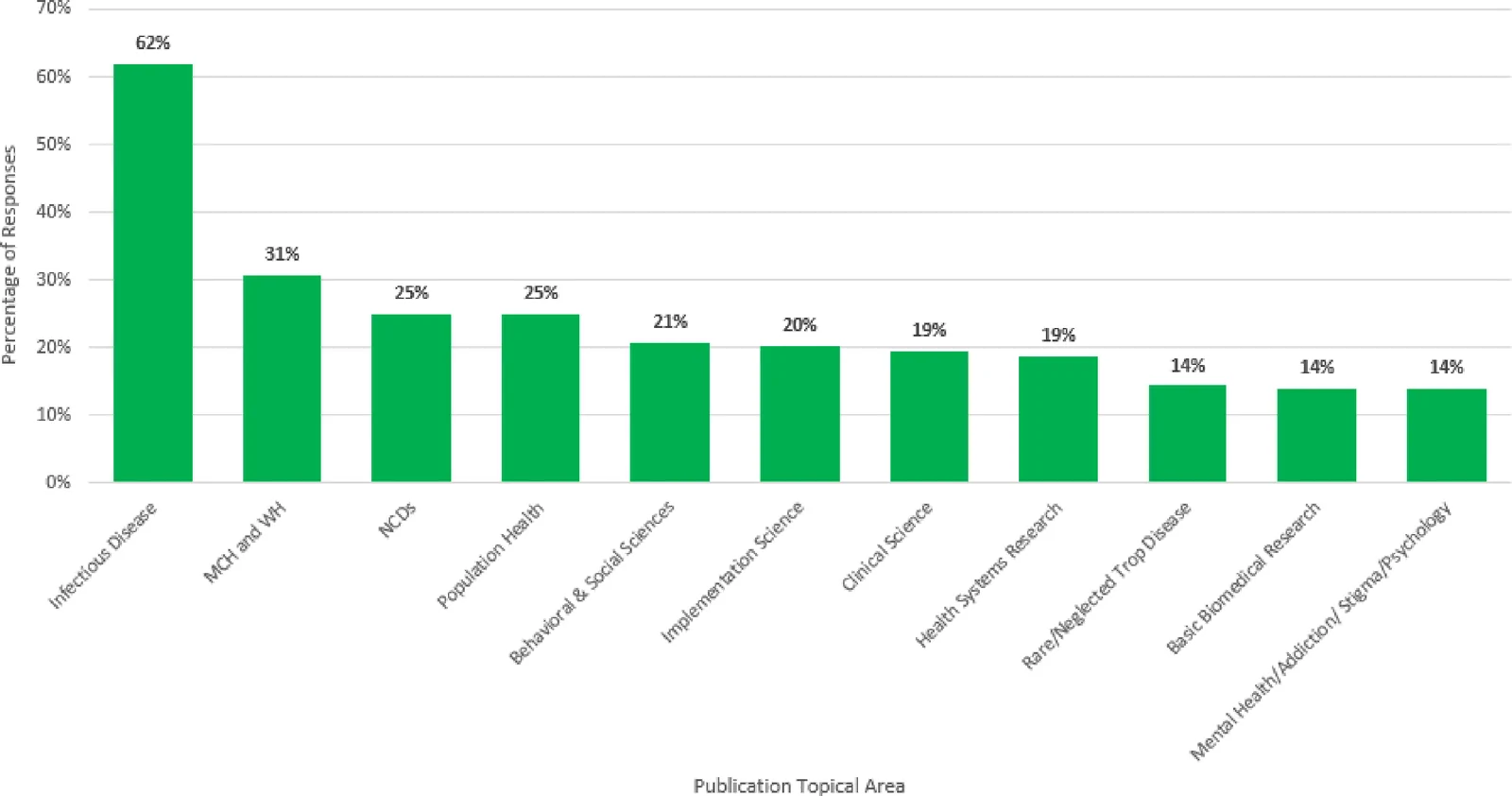
Topical area of alumni publications. *Note*: Topics less than 10% are removed.

A total of 67% of responding program alumni (450 of 673) applied for research funding after their F&S experience. This held true for both US and LMIC individuals (66% vs. 68%, respectively) and regardless of gender (69% for male and 66% for female respondents). Of the 450 who applied for funding, nearly half (47%) of the applications were sent to either Fogarty or another NIH institute. A total of 73% of all respondents (US and LMIC combined) reported in the survey that since their fellowship they have participated in international research. The survey also asked about publishing post-fellowship. A total of 605 survey respondents (90%) reported that they did publish after their training year ended. There was little difference when disaggregated by US versus LMIC trainee (91% vs. 88%, respectively) or gender (92% of male respondents published and 89% female). Of those publications, 90% were considered global health. Specific topics, as reported in the survey, focused on infectious diseases (62%) followed by topics related to maternal, child, and women’s health (31%) and then noncommunicable diseases (NCDs) and population health (both 25%; see Figure 3).

Lastly, survey participants were asked about their current employment. To allow for a minimum of five years post-fellowship to settle on a steady employment, Consortia II participants were removed from this data set leaving a total of 427 responses. When asked about their current employment, the majority of responding alumni reported going into the academic sector (n = 264; 62%; see Figure 4a). A total of 71% (303) respondents stated that their current employment is in the same topical area as their training research. The major emphasis of all types of employment was research (59%; 251), followed by clinical (24%; 100), and then teaching (15%; 62; see Figure 4b).

**Figure 4a:**
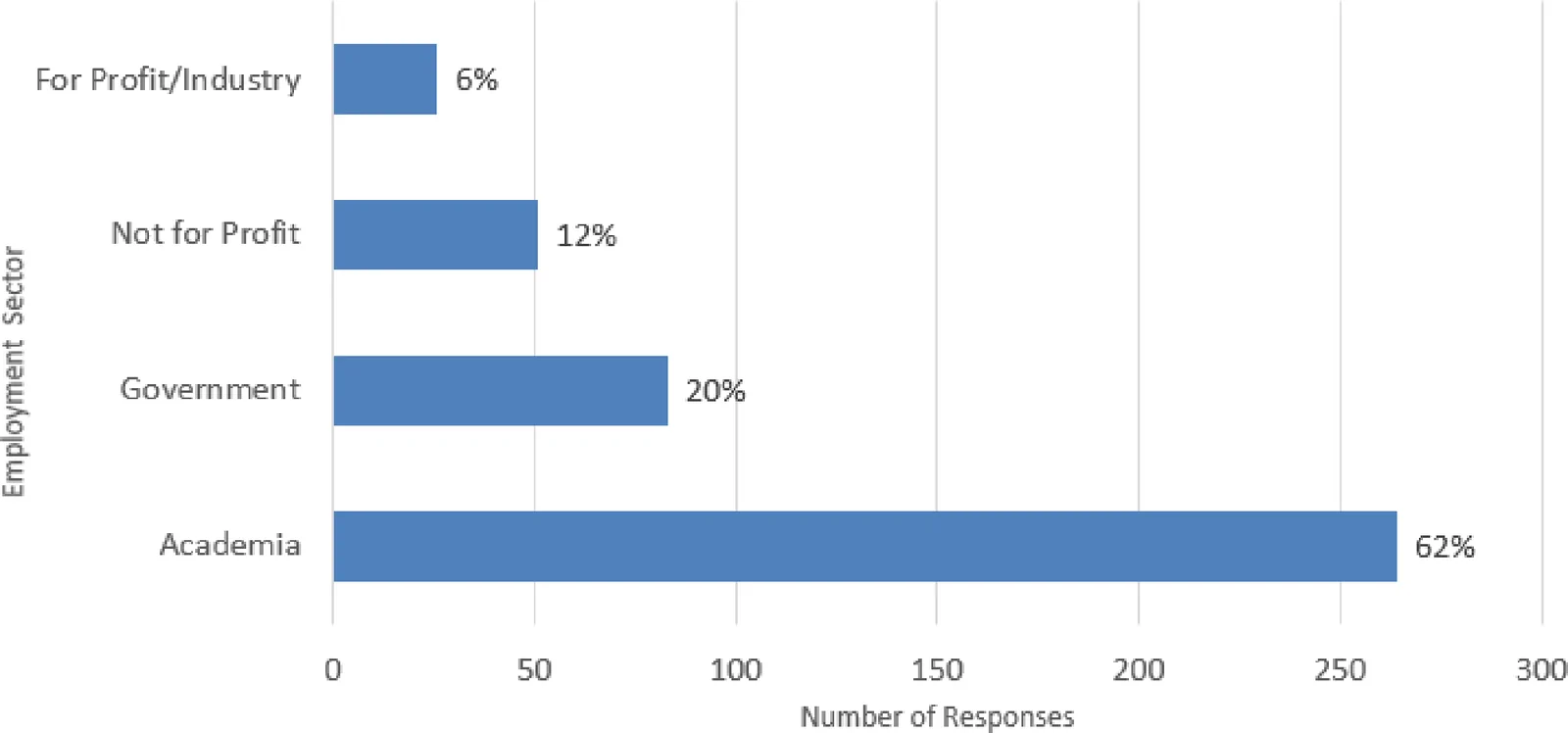
Alumni employment by sector.

**Figure 4b:**
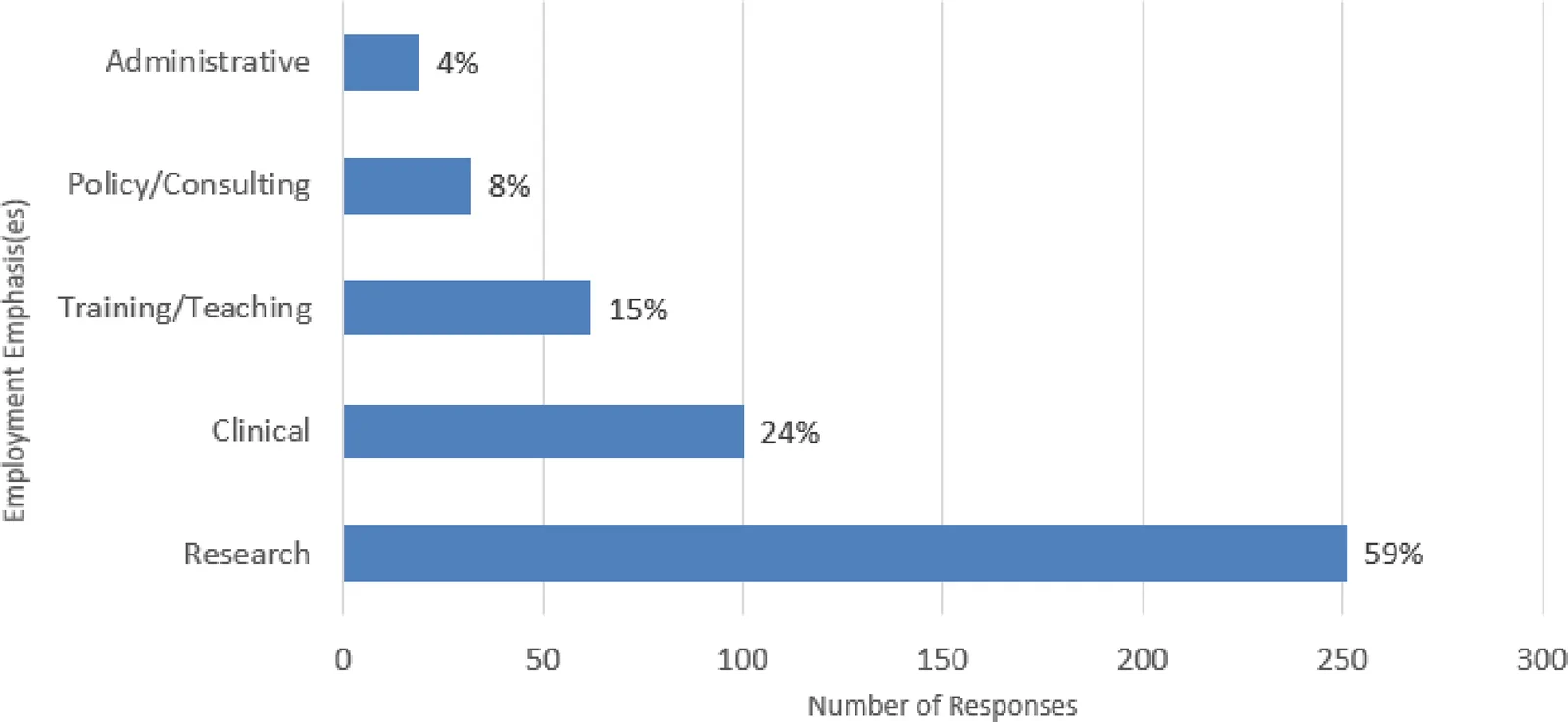
Alumni employment by emphasis.

## Conclusions

Over the past two decades, the Fogarty Global Health Program for Fellows and Scholars (F&S Program) has continuously evolved to strengthen its ability to develop the next generations of global health leaders. The program progressed from an internally administered Fogarty initiative serving primarily predoctoral trainees to a single grant mechanism supported by a coordinating center that managed fellowships for both predoctoral and postdoctoral researchers. Most recently, the program transitioned to a consortium model that engages a broader network of US institutions while expanding opportunities for trainees and mentors representing diverse scientific disciplines and research interests.

The program’s evolution paralleled broader shifts in the global health research landscape beyond its historical emphasis on infectious diseases. As NCDs—including cancer, cardiovascular disease and stroke, diabetes and obesity, mental health, and diseases associated with aging— assumed greater prominence in the field, the F&S Program likewise broadened its scientific scope. This transformation was accompanied by increased engagement of NIH ICOs, strengthened partnerships with LMIC institutions, and sustained growth in both the number and disciplinary diversity of trainees.

The expansion of eligibility to postdoctoral fellows and encouragement of applications from across the health sciences attracted greater engagement from NIH ICOs. Their financial support enabled the program to substantially increase trainee participation and recruit individuals from a broader range of scientific and professional backgrounds. This increase in disciplinary diversity, in turn, attracted engagement and support from additional NIH ICOs and necessitated a broader network of collaborating institutions and mentors across LMICs. As the scope of trainee research diversified, research areas that historically received limited attention in global health, such as mental health and other NCDs, became increasingly integrated into research agendas at partner institutions in LMICs. Collectively, the mutually reinforcing cycle of broader NIH ICO participation, disciplinary representation, and institutional invovement transformed the F&S Program into a more expansive, interdisciplinary, and globally connected training platform.

The F&S Program also served as a catalyst for expanding NIH engagement in global health research. Participation in the program enabled NIH ICOs, including those with previously limited investments in global health, to establish or broaden research and research training portfolios in areas such as cardiovascular disease, cancer, nephrology, and other specialty areas. Beyond supporting individual trainees, NIH ICO investments helped cultivate a pipeline of investigators with the skills, experience, and international collaborations needed to compete for subsequent NIH career development and independent research funding, including K- and R-series awards.

Survey findings indicate that many F&S alumni successfully transitioned to independent research careers, securing subsequent funding from NIH as well as other national and international funding organizations. These outcomes suggest that the program not only supported trainees during a formative stage of their careers but also contributed to the development of a sustainable global network of investigators. Alumni continued to collaborate on research, strengthen institutional partnerships, and build research capacity across LMICs. Such enduring international collaborations are essential for advancing scientific discovery while facilitating the translation of research into locally relevant policies, programs, and clinical practice. Consistent with these findings, many alumni reported maintaining both professional collaborations and personal relationships with colleagues and mentors from the United States and LMICs, underscoring the program’s lasting influence on global scientific partnerships.

These findings further demonstrate that mentored research training is an effective model for developing the global health research workforce. At the core of this model is high-quality mentorship, with trainees consistently identifying the mentoring relationship as the single most influential factor in the success of their fellowship experience. Mentors served not only as scientific advisors but also as role models for collaborative research, effective leadership, and ethical global health practice, inspiring many trainees to pursue independent careers in global health research. Yet mentorship operated within a broader enabling environment. Trainees emphasized the importance of a supportive research environment, including strong institutional infrastructure, access to interdisciplinary and international collaborations, peer support, protected time for research, and their own persistence and commitment. Together, these interconnected elements created an ecosystem that fostered scientific development, professional growth, and long-term career success.

For many fellows, participation in the F&S Program proved to be a defining experience that shaped the trajectory of their academic and professional careers. The program served as a critical bridge between formal degree training and independent research careers by providing protected time, structured mentorship, and opportunities to establish professional networks across US and LMIC institutions. For many participants, particularly those with limited prior exposure to global health, these experiences created lasting collaborations and expanded access to an international community of researchers.

By training nearly 1,500 fellows and scholars and engaging a broad network of mentors and institutional partners, the F&S Program strengthened research capacity and fostered enduring partnerships between US and LMIC institutions. Equally important, it inspired early-career scientists and public health professionals to pursue global health research aligned with their interests by providing immersive, mentored research experiences that demonstrated the opportunities and challenges of a career in global health research. It also established the professional relationships, perspectives, and collaborative networks that continued to shape their careers long after completion of the fellowship.

The long-term influence of the F&S Program is reflected in the career trajectories of its alumni and the institutional capacity it helped build. Many alumni transitioned to independent research careers, secured subsequent research funding, and remained engaged in global health research, demonstrating the program’s sustained impact on workforce development. As interest in global health research among US health sciences trainees has grown, the program has contributed to the expansion of global health programs at US academic institutions by helping develop a cadre of faculty with international research experience and by broadening institutional research portfolios over time. Beyond individual career development, the program strengthened the broader global health research ecosystem. Organizations such as the Consortium of Universities for Global Health have provided an enduring professional community for participating institutions and alumni, while partnerships with LMIC institutions have enhanced local research capacity, strengthened institutional relationships, and created opportunities to train the next generations of investigators. Collectively, these findings demonstrate that investments in mentored, partnership-based global health research training can generate lasting benefits for individuals, institutions, and the global scientific community, offering a model for future programs seeking to strengthen the global health research workforce and advance equitable international research partnerships.

Several limitations should be considered when interpreting these findings. First, the evaluation relied primarily on cross-sectional survey data and lacked pre- and post-program measures, limiting the ability to attribute observed career outcomes directly to participation in the F&S Program. Response bias is also possible, as the two cross-sectional surveys differed in design (one anonymous and one non-anonymous), respondents were not necessarily the same individuals, and the moderate response rates may have disproportionately captured alumni who maintained stronger connections to the program or remained engaged in global health research. Consequently, the findings may overestimate program effectiveness and are subject to selection bias.

Second, the absence of a comparison group precludes assessment of the counterfactual—whether participants would have achieved similar career outcomes in the absence of the program. As a result, the factors identified by participants as contributing to their success, while informative, should not be interpreted as evidence of causal relationships or the relative effectiveness of this training model compared with alternative approaches. Future evaluations would benefit from longitudinal study designs, standardized outcome measures, and comparative analyses across global health research training programs to strengthen inferences regarding the program’s contribution to researcher independence, sustained engagement in global health science, and long-term institutional capacity building.

Despite these limitations, the findings are consistent with prior evaluations demonstrating the importance of mentorship, immersive international research experiences, and sustained institutional partnerships in fostering careers in global health research [17–19]. As one of the largest programs to provide year-long, mentored research experiences for predoctoral and postdoctoral trainees in LMIC settings, the F&S Program offers a valuable model for developing the global health research workforce through collaborative, partnership-based training. Its evolution over two decades illustrates how sustained investments in mentorship, international collaboration, and institutional capacity can cultivate a diverse pipeline of researchers prepared to address increasingly complex global health challenges. Ultimately, the program underscores that advancing global health requires not only scientific excellence but also enduring partnerships that transcend institutional and national boundaries, recognizing that many of today’s most pressing health challenges are inherently global and require collaborative solutions.
